# Twitter users perceptions of AI-based e-learning technologies

**DOI:** 10.1038/s41598-024-56284-y

**Published:** 2024-03-11

**Authors:** Luisa Stracqualursi, Patrizia Agati

**Affiliations:** Department of Statistics, University of Bologna, 40126 Bologna, Italy

**Keywords:** Human behaviour, Machine learning, Psychology and behaviour, Human behaviour, Machine learning, Psychology and behaviour

## Abstract

Today, teaching and learning paths increasingly intersect with technologies powered by emerging artificial intelligence (AI).This work analyses public opinions and sentiments about AI applications that affect e-learning, such as ChatGPT, virtual and augmented reality, microlearning, mobile learning, adaptive learning, and gamification. The way people perceive technologies fuelled by artificial intelligence can be tracked in real time in microblog messages promptly shared by Twitter users, who currently constitute a large and ever-increasing number of individuals. The observation period was from November 30, 2022, the date on which ChatGPT was launched, to March 31, 2023. A two-step sentiment analysis was performed on the collected English-language tweets to determine the overall sentiments and emotions. A latent Dirichlet allocation model was built to identify commonly discussed topics in tweets. The results show that the majority of opinions are positive. Among the eight emotions of the *Syuzhet* package, ‘trust’ and ‘joy’ are the most common positive emotions observed in the tweets, while ‘fear’ is the most common negative emotion. Among the most discussed topics with a negative outlook, two particular aspects of fear are identified: an ‘apocalyptic-fear’ that artificial intelligence could lead the end of humankind, and a fear for the ‘future of artistic and intellectual jobs’ as AI could not only destroy human art and creativity but also make the individual contributions of students and researchers not assessable. On the other hand, among the topics with a positive outlook, trust and hope in AI tools for improving efficiency in jobs and the educational world are identified. Overall, the results suggest that AI will play a significant role in the future of the world and education, but it is important to consider the potential ethical and social implications of this technology. By leveraging the positive aspects of AI while addressing these concerns, the education system can unlock the full potential of this emerging technology and provide a better learning experience for students.

## Introduction

### AI-powered e-learning technologies

Current technology continues to advance, continuously transforming the way we live and the way we learn. Over the past few years, e-learning has become increasingly popular, as more people turn to online platforms for education and training. The COVID-19 pandemic has further accelerated this trend, as traditional classroom-based learning has become difficult, if not impossible, in many parts of the world.

Some of the key trends that we are also likely to see in the future of e-learning are as follows:

- *Adaptive learning.* This method uses AI technology to personalize the learning experience for each student. This method adjusts the presentation of educational material according to an individual learner’s needs, preferences, and progress. By analysing students’ performance, it is possible to track their progress, and identify areas where they need more support or challenge.^1–3^
- *Immersive learning.* This refers to an educational approach that involves deeply engaging learners in a simulated or interactive environment. It seeks to create a sense of immersion, in which individuals feel fully involved in the learning process through various sensory experiences. The primary goal is to enhance the understanding, retention, and application of knowledge or skills. The key components of immersive learning include virtual and augmented reality (VR/AR). These technologies are becoming increasingly advanced and accessible, and we are likely to see more e-learning platforms using these technologies.^4,5^ For example, a medical student could use VR to practice surgical techniques in a simulated operating room,^6^ or an engineering student could use AR to visualize complex machinery and processes.^7^
- *Microlearning.* This refers to short, bite-sized learning modules that are designed to be completed in a few minutes or less. These modules are ideal for learners who have limited time or attention spans, and they can be easily accessed on mobile devices. In the future, we are likely to see more e-learning platforms using microlearning to deliver targeted, on-demand learning experiences.^8,9^
- *Gamification.* This refers to the use of game-like elements, such as badges, points, and leaderboards, to increase engagement and motivation among learners. Through the addition of game-like features to e-learning courses, learners can be incentivized to complete assignments and reach learning goals; thus, the learning experience becomes more engaging and enjoyable.^10–12^
- *Mobile learning.* With the widespread use of smartphones and tablets, e-learning is becoming more mobile-friendly, allowing learners to access course materials and complete assignments on the go. This makes learning more convenient and accessible, as learners can fit their learning into their busy schedules and on-the-go lifestyles.^13,14^
- *Social learning.* Social media and collaborative tools can enable learners to connect and learn from each other in online communities.^15^ Learners can share their experiences, ask questions, and receive feedback from their peers, creating a sense of community and collaboration that can enhance the learning experience.^16^
- *Generative AI.* This refers to a subset of artificial intelligence focused on creating new content or information. It involves algorithms and models that are capable of generating novel content that can resemble human-generaed data. In February, 2022, an AI generative system named *AlphaCode* was launched. This system has been trained to ‘understand’ natural language, design algorithms to solve problems, and then implement them in code. At the end of November 2022, a new generative artificial intelligence chatbot that was developed by OpenAI and named *ChatGPT* was launched. ChatGPT has a wide range of potential applications due to its ability to generate human-like responses to natural language input. Some of its potentialities include text generation, summarization of long texts into shorter summaries, language translation, question answering, text completion, text correction, programming code generation, equation solutions and so on. ChatGPT evolved into OpenAI’s most widely used product to date, leading to the launch of ChatGPT Plus, a pilot paid subscription, in March 2023. Overall, the potential of AlphaCode and ChatGPT are vast, and these tools are likely to be used in many applications in the future of e-learning, as their capabilities will continue to improve through further research and development. ChatGPT can be integrated as a conversational AI within e-learning platforms. It can provide real-time responses to queries, clarify doubts, offer explanations, and guide learners through course materials. It is a promising tool for language lessons since it can translate text from one language to another.^17^ To assist students and improve their writing abilities, ChatGPT may check for grammatical and structural problems in their work and provide valuable comments.^18^ Students can explore many things with the help of ChatGPT, such as developing a computer program, writing an essay and solving a mathematical problem.^19^ AlphaCode can aid e-learning platforms focused on programming or coding courses. It can provide code suggestions, explanations, and debugging assistance, helping learners better understand coding concepts.^20^

### How people perceive AI-based technologies: general and e-learning-focused literature overview

In the literature, people’s perceptions of technologies fuelled by artificial intelligence (AI) can vary depending on various factors such as their personal experiences and cultural backgrounds, and the way in which AI is portrayed in the media. The following are some common perceptions of AI technologies:

- *Fear of Job Loss.* One of the most common fears associated with AI technologies is that they will take over jobs previously performed by humans. This fear is especially prominent in industries such as manufacturing, customer service, translation^21–23^ and teaching^24^.
- *Improved Efficiency.* Many people view AI technologies as a way to improve efficiency and accuracy in various fields. For example, AI-powered tools can improve students’ performance,^25^ AI-powered software can help doctors diagnose diseases,^26^ and chatbots can help students^27^ and customer service representatives answer queries more quickly.^28^
- *Ethical Concerns.* There are concerns about the ethical implications of AI, such as bias in decision-making,^29^ invasion of privacy,^30^ and the potential for the development of AI-powered weapons.^31^ For example, schools and institutions use AI-powered technologies to analyse student academic performance and collect a large amount of personal identity data; if these data are leaked or misused, this will seriously affect students’ personal privacy and security. In addition, students have difficulty controlling their own data and understanding how it is being used and shared, which may also lead to concerns and mistrust regarding personal privacy.^32,33^
- *Excitement for Innovation.* Some people are excited about the potential of AI to bring about new and innovative solutions to long-standing problems. For example, AI is being used to develop autonomous vehicles, which could revolutionize transportation,^34^ and new methods for teaching and learning music.^35^
- *Lack of Trust.* Many people are still sceptical about the reliability and safety of AI technologies, especially given recent high-profile incidents of AI systems making mistakes^36^ or being manipulated.^37^ The lack of trust in the current application of generative AI in education mainly involves two aspects: opacity and reliability. When AI gives a result, it is difficult to explain the decision-making process, which makes it difficult for students to understand why they obtain a particular answer and how to improve their mistakes (opacity). Moreover, generative AI needs to be trained on a large dataset to ensure its effectiveness and reliability. For example, to train an effective automatic grading model, a large dataset of student essays and high-quality labelled data, such as scores for grammar, spelling, and logic, is needed. Insufficient datasets or low-quality labelled data may cause an automatic grading model to make mistakes and miss important aspects and affect its accuracy and application effectiveness.^38^

Overall, people’s perceptions of AI technologies are complex and multifaceted, are influenced by a range of factors and are likely to continue evolving as AI becomes more integrated into our lives.

### Purpose and outline of the paper

Considering what has been said thus far, it could be interesting to explore sentiments and major topics in the tweets about the new AI-based technologies.

Social media are indeed a major and rich data source for research in many domains due to their 4.8 billion active users^39^ across the globe. For instance, researchers analyse user comments extracted from social media platforms (such as Facebook,^40^ Twitter,^40^ and Instagram^41^) to uncover insights into social issues such as health, politics and business. Among these platforms, Twitter is one of the most immediate; tweets flow nonstop on the bulletin boards of users. Twitter allows users to express and spread opinions, thoughts and emotions as concisely and quickly as possible. Therefore, researchers have often preferred to analyse user comments on Twitter to immediately uncover insights into social issues during the COVID-19 pandemic (e.g., conspiracy theories,^42^ why people oppose wearing a mask,^43^ experiences in health care^44^ and vaccinations^45^) or distance learning.^46–48^

Furthermore, we chose Twitter for its ability to immediately capture and spread people’s opinions and emotions on any topic, as well as for its ability to provide plentiful data, even in a short amount of time. Moreover, the people who have more direct experience with e-learning and AI technologies are students, teachers and researchers, i.e., persons of school or working age; that is, people who, by age, make up approximately 83% of Twitter users.^49^

The text content of a tweet is a short microblog message containing at most 280 characters. This feature makes tweets particularly suitable for natural language processing (NLP) techniques, which are widely used to extract insights from unstructured texts and can then be used to explore sentiments and major topics of tweets. Unlike traditional methods, which use surveys and samples to evaluate these frameworks and are expensive and time-consuming, NLP techniques are economical and fast and provide immediate results.

In this paper, we aim to answer three main questions related to the first months following the launch of ChatGPT:

- What has been the dominant sentiment towards AI-powered technologies? We responded through a sentiment analysis of related tweets. We used VADER as a sentiment analysis tool.^50^
- Which emotions about AI-powered technologies are prevalent? In this regard, we explored the emotions to the tweets using the *Syuzhet* package.^51^
- What are the most discussed topics among those who have positive feelings and those who have negative feelings? With respect to this problem, we used the latent Dirichlet allocation (LDA) model.^52^

The findings from this study could aid in reimagining education in the postpandemic era to exploit technology and emerging strategies as benefits for educational institutions rather than as preparation for a new possible increase in infections. To this end, we decided to use only the technologies listed in “AI-powered e-learning technologies” as keywords for extracting tweets.

## Methodology

### The data

Twitter was chosen as the data source. It is one of the world’s major social media platforms, with 237.8 million active users in July 2022,^53^ and it is also a common source of text for sentiment analyses.^54–56^

To collect AI-related tweets, we used ‘Academic Account for Twitter API V2’, which provides historical data and allows for the data to be filtered by language and geolocation.^57^

For our study, we chose geolocated English-tweets only, posted from November 30, 2022 - March 31, 2023, with one or more of the following keywords: ‘ChatGPT’, ‘AlphaCode’, ‘virtual reality’, ‘augmented reality’, ‘micro-learning’, ‘mobile learning’, ‘adaptive learning’, ‘social leaning’, ‘AI’, ‘AI learning’ and ‘gamification’. A total of 31,147 tweets were collected.

### Data preprocessing

In order to prepare the data for sentiment analysis, we employed various preprocessing techniques using NLP tools in Python. The steps we followed are as follows:

1. Eliminated mentions, URLs, and hashtags from the text,
2. Substituted HTML characters with their respective Unicode equivalents (e.g., replacing ‘ &amp;’ with ‘ &’),
3. Removed HTML tags such as<br>,<p>, and others,
4. Eliminated unnecessary line breaks,
5. Removed special characters and punctuation except for exclamation points (the exclamation point is the only punctuation marks to which the used VADER lexicon is sensitive),
6. Excluded words that consist of only numbers.

For the second part, a high-quality dataset was required for the topic model. To achieve this, we removed duplicate tweets. In addition to the general data cleaning methods, we employed tokenization and lemmatization techniques to enhance the model’s performance.

We used the *Gensim *library^58^ to tokenize the text, converting all the content to lowercase to ensure uniformity in word representation. Next, we pruned the vocabulary by removing stop words and terms unrelated to the topic. Additionally, we created a ‘*bigrams*' model to capture meaningful word combinations.

Finally, we employed the ‘*spaCy*' library from NLTK^59^ to carry out lemmatization, which helped simplify words to their base form.

### Sentiment and emotions analysis

To conduct sentiment analysis, we utilized the Valence Aware Dictionary for Sentiment Reasoning (VADER) algorithm, developed by Hutto et al.^50^ VADER is a sentiment analysis tool that uses a sentiment lexicon, a dictionary specifically designed for sentiment analysis, to determine the emotion intensity of sentiment expressed in a text. The lexicon consists of words or phrases with their accompanying sentiment ratings. It allows for efficient sentiment analysis of social media content and exhibits remarkable accuracy comparable to that of humans.

**Figure 1:**
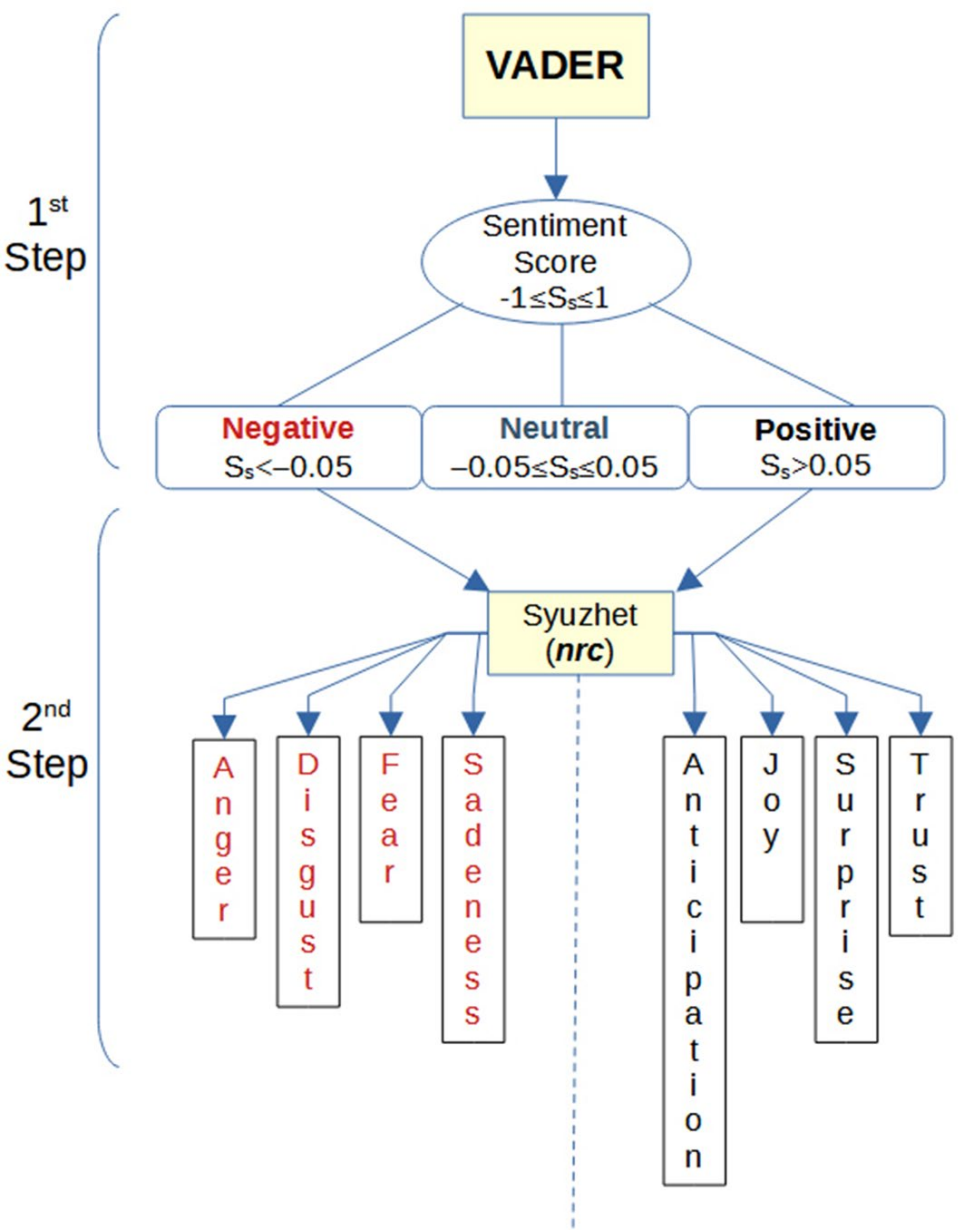
Steps in determining sentiment and emotions analysis.

Using VADER, we assigned sentiment scores to the preprocessed text data of each tweet. We followed the classification method recommended by the authors, categorizing the sentiment scores into three main categories: positive, negative, and neutral (see Fig. 1—1st Step).

VADER has demonstrated outstanding performance in analysing social media text. Comprehensive rules to consider various lexical features, including punctuation, capitalization, degree modifiers, the contrastive conjunction ’but’ and negation flipping trigrams.

Next, we employed the ‘*nrc*' algorithm, a component of the R library *Syuzhet* package,^51^ to explore the underlying emotions associated with the tweet categories. In the ‘*nrc*' algorithm, an emotion dictionary is utilized to evaluate each tweet based on two sentiments (positive or negative) and eight emotions (anger, fear, anticipation, trust, surprise, sadness, joy, and disgust). Its purpose is to recognize the emotions conveyed within a tweet.

Whenever a tweet is connected to a specific emotion or sentiment, it receives points indicating the degree of valence in relation to that category. For instance, if a tweet includes three words associated with the ‘fear’ emotion in the word list, the tweet will receive a score of 3 in the fear category. Conversely, if a tweet does not contain any words related to a particular emotion, it will not receive a score for that specific emotion category.

When employing the ‘*nrc*’ lexicon, each tweet is assigned a score for each emotion category instead of a single algebraic score based on positive and negative words. However, this algorithm has limitations in accounting for negators and relies on a bag-of-words approach, disregarding the influence of syntax and grammar. Consequently, the VADER and ‘*nrc*’ methods are not directly comparable in terms of tweet volume and polarity categories.

Therefore, we utilized VADER for sentiment analysis and subsequently employed the ‘*nrc*' algorithm specifically for identifying positive and negative emotions. The sentiment analysis process follows a two-step procedure, as illustrated in Fig. 1. While VADER’s neutral tweets play a valuable role in classification, they are not particularly informative for emotion analysis. Therefore we focused on tweets exhibiting positive and negative sentiments. This methodology was the original source of our previous paper.^60^

### The topic model

The topic model is an unsupervised machine learning method; that is, it is a text mining procedure that can be used to identify the topics or themes of documents in a large document corpus.^61^ The latent Dirichlet allocation (LDA) model is one of the most popular topic modelling methods; it is a probabilistic model for expressing a corpus based on a three-level hierarchical Bayesian model. Latent Dirichlet allocation (LDA) is a generative probabilistic model of a corpus. The basic idea is that documents are represented as random mixtures over latent topics, where each topic is characterized by a distribution over words.^62^ Particularly in LDA models, the generation of documents within a corpus follows the following process:

1. A mixture of *k* topics, $\theta$, is sampled from a Dirichlet prior, which is parameterized by $\alpha$;
2. A topic $z_n$ is sampled from the multinomial distribution, $p(\theta \mid \alpha )$ that is the document topic distribution which models $p(z_{n}=i\mid \theta )$ ;
3. Fixed the number of topics $k=1...,K$, the distribution of words for *k* topics is denoted by $\phi$ ,which is also a multinomial distribution whose hyper-parameter $\beta$ follows the Dirichlet distribution;
4. Given the topic $z_n$, a word, $w_n$, is then sampled via the multinomial distribution $p(w \mid z_{n};\beta )$.

Overall, the probability of a document (or tweet, in our case) “$\textbf{w}$ ” containing words can be described as:

1 $$\begin{aligned} p(\textbf{w})=\int _\theta {p(\theta \mid \alpha )\left( {\prod \limits _{n = 1}^N {\sum \limits _{z_n = 1}^k {p(w_n \mid z_n;\beta )p(z_n \mid \theta )} } } \right) } \mathrm{}d\theta \end{aligned}$$

Finally, the probability of the corpus of *M* documents $D=\{\textbf{ w}_{\textbf{1}},...,{\textbf{w}}_{\textbf{M}}\}$ can be expressed as the product of the marginal probabilities of each single document $D_m$, as shown in (2).

2 $$\begin{aligned} p(D) = \prod \limits _{m = 1}^M {\int _\theta {p(\theta _m \mid \alpha )\left( {\prod \limits _{n = 1}^{N_m } {\sum \limits _{z_n = 1}^k {p(w_{m,n} \mid z_{m,n};\beta )p(z_{m,n} \mid \theta _m )} } } \right) } } \mathrm{}d\theta _m \end{aligned}$$

An essential challenge in LDA is deterining an appropriate number of topics. Roder et al.^63^ proposed coherence scores to evaluate the quality of each topic model. In particular, topic coherence is the metric used to evaluate the coherence between topics inferred by a model. As coherence measures, we used $C_v$ which is a measure based on a sliding window that uses normalized pointwise mutual information (NPMI) and cosine similarity.^63^ This value is used to emulate the relative score that a human is likely to assign to a topic and indicate how much the topic words ‘make sense’. This score is used to infer cohesiveness between ‘top’ words within a given topic. For topic visualization we used PyLDAvis, a web-based interactive visualization package that facilitates the display of the topics that were identified using the LDA approach.^52^ In this package, each topic is visualized as a circle in a two-dimensional plane determined by principal components between topics and used Multidimensional scaling is used to project all the interrelated topic distances to two dimensions.^64^ In the best hypothetic situation, the circles have similar dimensions and are well spaced from each other, covering the entire space made up of the 4 quadrants of the graph. An LDA model is evaluated the better the more the coherence is and the closer the pyLDAvis visualization is to the hypothetical situation.

## Results

### Exploring the tweets

**Figure 2:**
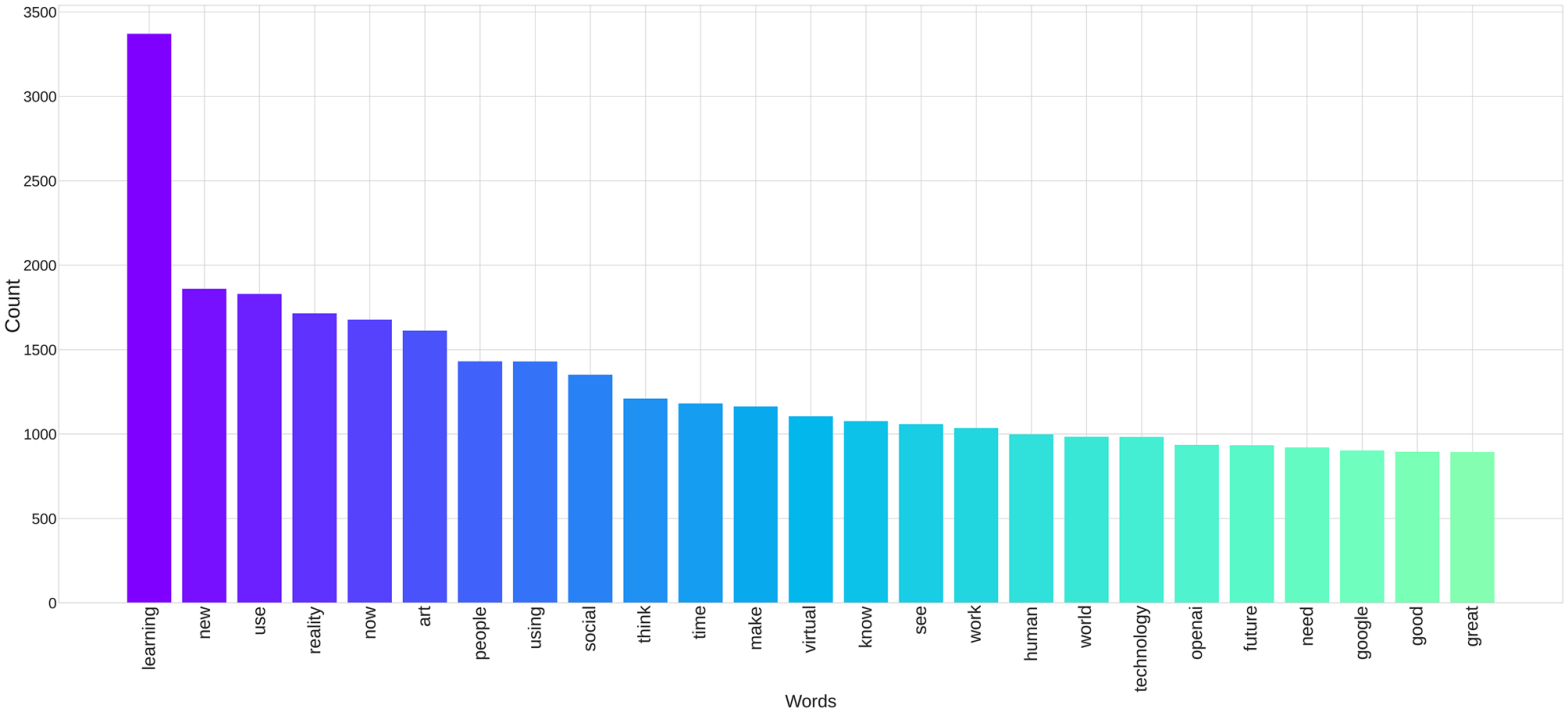
The total text word frequency. After removing irrelevant words, we counted and visualized the 25 most frequent words in our dataset.

The word frequency of the most frequent 25 words terms are counted and visualized in Fig. 2. The words are related to new AI tools and have positive attributes such as ‘good’ or ‘great’.

**Figure 3:**
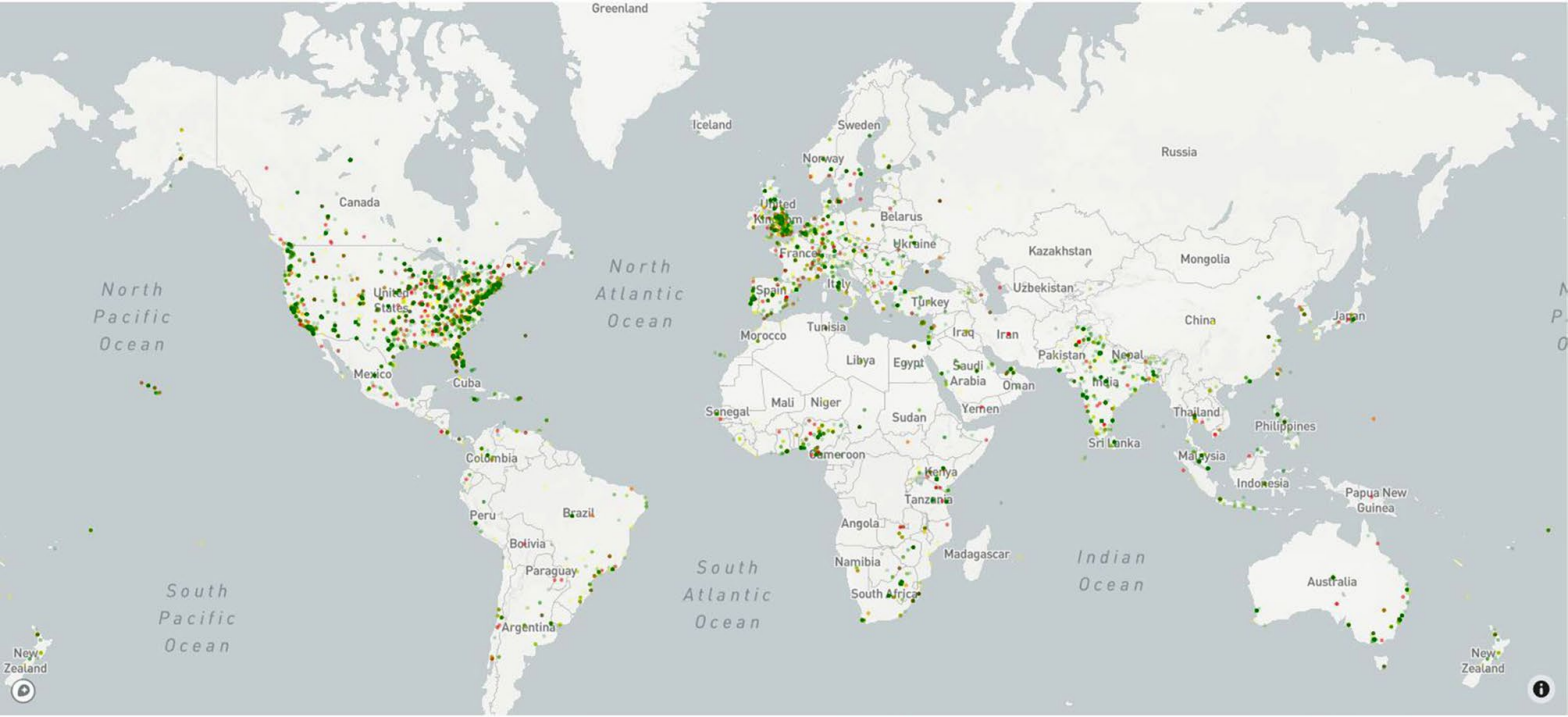
Tweets according to polarity by country.

All the tweets extracted were geolocated, but the ‘user location’ was detected in only approximately 85% of the tweets, highlighting the number of tweets that came from different counties around the world. The countries with the highest number of tweets are the United States and Great Britain (Fig. 3) but this result should be read considering that we have extracted tweets in the English language; therefore, it is normal that in countries where English is spoken, the number of tweets is very high. Notably, India and Europe also have a many Twitter users. In the United States, most Twitter users are located near the East and West Coasts. This figure also shows the polarity of the tweets: the colours red, yellow and green indicate negative, neutral and positive tweets, respectively.

### Sentiment analysis

The sentiment score of a sentence is calculated by summing up the lexicon rates of each VADER-dictionary-listed word in the sentence. After proper normalization is applied, VADER returns a ‘compound’ sentiment score ($S_s$) in the range of $-1$ to 1, from the most negative to the most positive. Once the score $S_s$ is known, threshold values can be used to categorize tweets as positive, negative, or neutral (see Fig. 1—1st Step). According to our analysis, the output of the VADER model shows a great predominance of positive public opinion (Table 1). As an example, three tweets with their own polarity are shown in Table 2. Table 3 shows the number of total tweets with the related percentages and the percentages of positive, negative and neutral tweets of the various AI applications examined.

**Table 1:** Number of tweets for sentiment polarity (SP).

SP	Absolute values	Percentages
Positive	17933	57.58
Negative	5185	16.65
Neutral	8029	25,78

**Table 2:** Some examples of tweets with a sentiment score (${{S}}_s$ ) and polarity .

Polarity	${{{S}}}_s$	Text
Negative	$-$ 0.9607	If artificial intelligence is used to cheat on tests it serves no purpose the individual using it remains stupid, a grade of A means nothing if you have no knowledge about the job you’re applying for dumb remains dumb by cheating your even dumber! And should be held liable for it
Neutral	0	ML handling structured and semi-structured data. AI is a process of extracting insights from unstructured data. Deep learning deals with both structured, semi-structured, and unstructured data to train a neural network
Positive	0.9776	Been busy this week @ErwinMiddle filming some hopefully exciting PD videos on #gamification! The beginning video was a bit rough but they are getting better lol. Hey I’m learning too! Thanks to our awesome leadership for allowing me the space to explore my passion!

**Table 3:** Results of sentiment analysis for tweet keywords.

Tweet keywords	$n_{tot}$	$n_{tot}$(%)	% pos	% neg	% neu
AI	15497	49.75	60.44	17.21	22.35
AI learning	1318	4.23	65.71	14.11	20.18
AR	595	1.91	63.36	8.74	27.90
AlphaCode	31	0.10	45.16	22.58	32.26
ChatGPT	11052	35.48	50.01	16.98	33.01
VR	957	3.07	59.67	15.67	24.66
Adaptive learning	54	0.17	74.07	12.96	12.96
Gamification	302	0.97	69.54	13.91	16.56
Microlearning	23	0.07	60.87	13.04	26.09
Mobile learning	137	0.44	59.12	12.41	28.47
Social learning	1181	3.79	73.41	14.99	11.60

The columns after the first indicate the number of total tweets, their relative percentages, and the percentage of positive, negative, and neutral tweets in each category, respectively.

**Figure 4:**
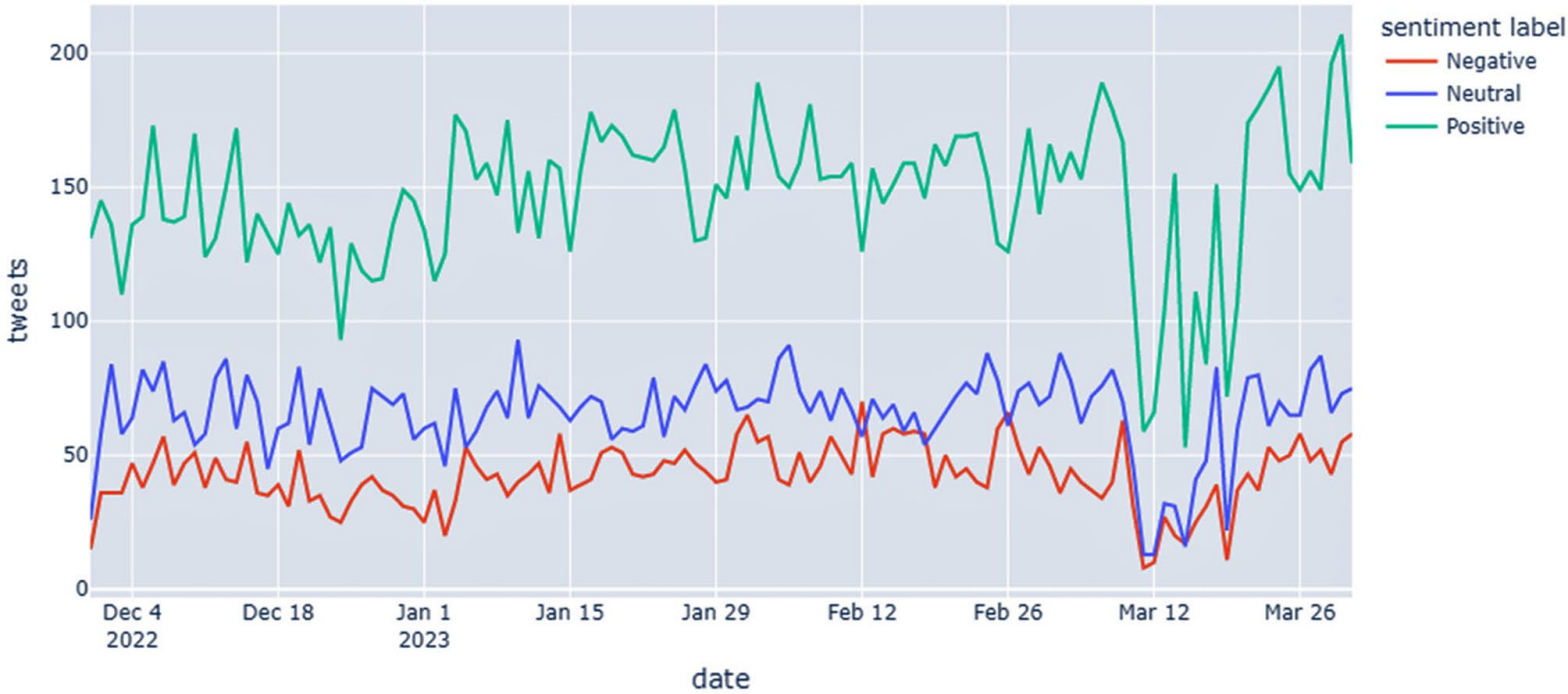
Timeline showing the sentiment of tweets.

Regarding the timeline, the results showed that the number of tweets slowly increased during the observation period (Fig. 4). Clearly, as shown in the chart, there was a single weekly decline in the third week of March, likely due to the St. Patrick’s Day holiday, which fell close to the weekend (March 17).

**Figure 5:**
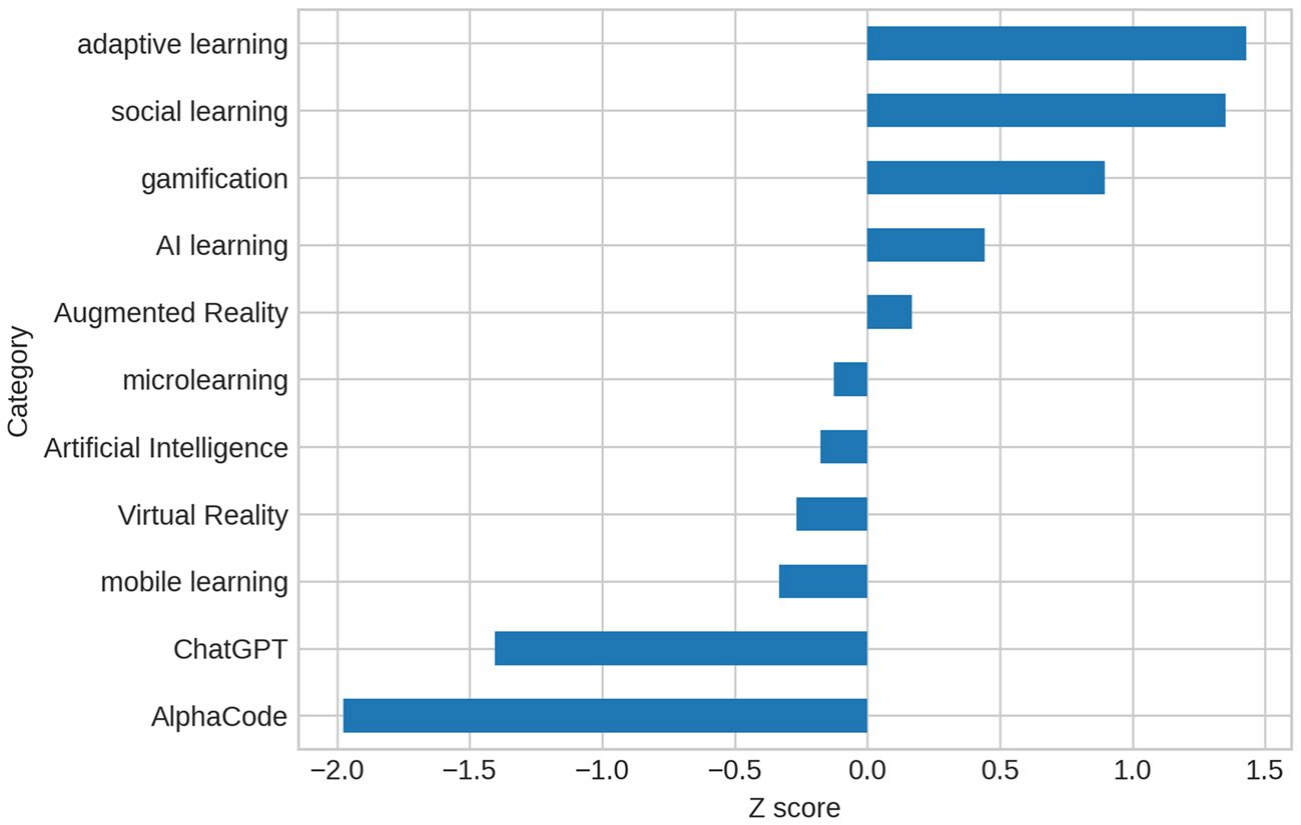
*z* scores of relative frequencies for positive sentiments.

The graph in Fig. 5 includes only the tweets with positive sentiment towards the 11 AI-based tools (categories). It returns the *z* scores of the relative frequencies $Fr_{i}$ of positive tweets of the *i*-tool ($i=1,...,11$), where the *z* score is computed as follows:

$$\begin{aligned} z_{i}=\frac{Fr_{i}-M(Fr_{})}{S(Fr_{})} { \ } \end{aligned}$$

A *z* score describes the position of a score (in our case $Fr_i$) in terms of standard deviation units from the average. This score is grater then 0 if the relative frequency of $Fr_i$ lies above the mean, while it is less than 0 if $Fr_i$ lies below the mean.

We preferred z scores to relative frequencies because the different categories have different averages: the z scores highlight the position of each $Fr_i$ with respect to the average of all the relative frequencies.

In Fig. 5, we can see that the new generative AI ‘AlphaCode’ and ‘ChatGPT’ have scores that are much lower than and far from the average of positive sentiments. This could be due to concerns about the possible errors of such AI-based tools. Instead, ‘adaptive learning’, ‘social learning’ and ‘gamification’ lie above the mean of positive sentiments. This clearly attests to the more positive sentiment towards these tools, in our opinion largely due to their immediate feedback and to their attitude towards keeping learners engaged and motivated.

**Figure 6:**
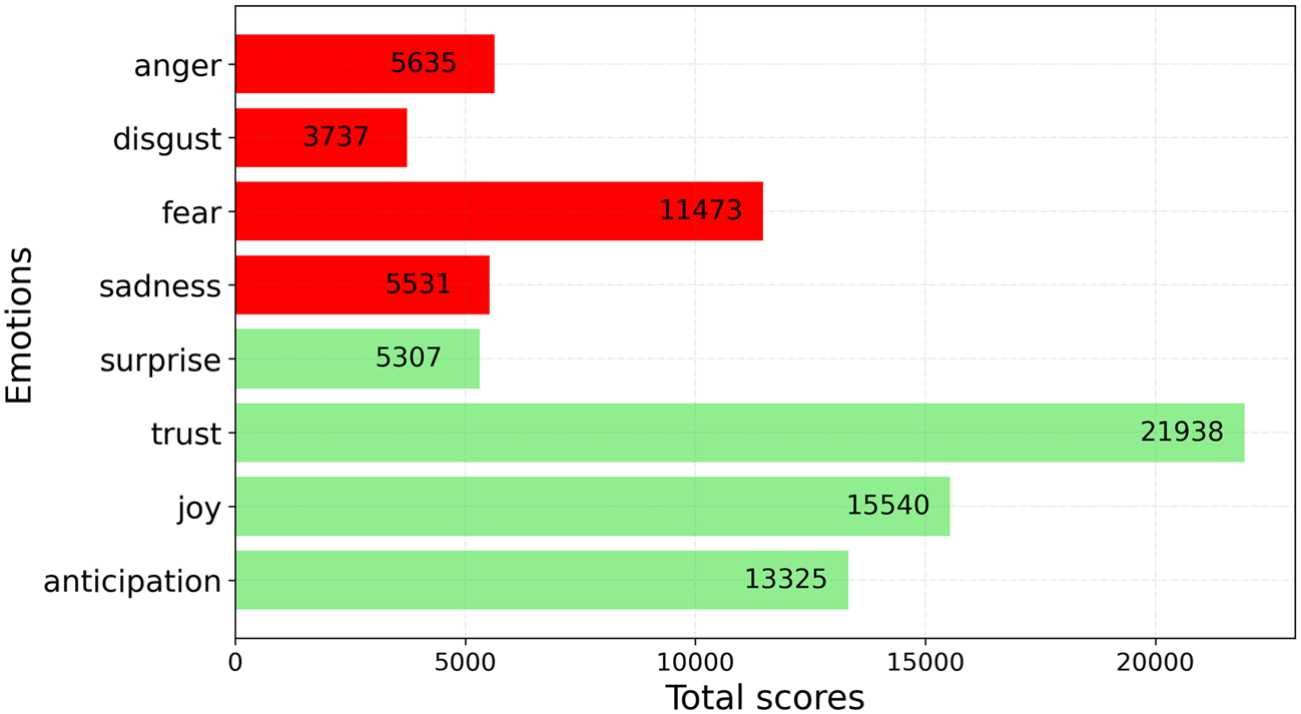
Emotion analysis of non-neutral tweets performed by *Syuzhet*.

The second step of the analysis focused on identifying emotions in non-neutral tweets (see Fig. 1—2nd Step). Among the eight basic emotions, ‘trust’ was the most common positive emotion observed in the tweets, followed by ‘joy’, while ‘fear’ was the most common negative emotion (Fig. 6). These results need to be interpreted in light of recent literature on the psychological dimensions of AI-based e-learning technologies (see section "How people perceive AI-based technologies: general and e-learning-focused literature overview"). In the literature, the dimension of fear includes the fear of job loss for teachers but also for the entire working world,^21–23,65^ as well as concerns about AI systems making mistakes^36^ or being manipulated.^37^ The ‘trust’ dimension could be interpreted as the expectation that such technologies can improve the performance of students and people in general,^26^ while ‘joy’ could be associated with enthusiasm for the potential of artificial intelligence in creating new and innovative solutions.^34^

### The topic model

To explore what concerns about AI-based tools Twitter users have, we applied the LDA model to our clean corpus of 28,259 words, which included only the following tagger components: nouns, adjectives, verbs and adverbs. Our goal was not to discover the topics discussed in the whole set of tweets but to detect the topics discussed in the positive sentiment tweets and the topics discussed in the negative sentiment tweets. Due to the large difference between the number of tweets with positive and negative sentiment polarity (57.58% vs. 16.65%), the application of the LDA model to the whole dataset would lead to not seeing the topics discussed in tweets with negative sentiment. Therefore, we chose to create two LDA models: one for tweets with positive polarity and one for those with negative polarity. For a better representation of the entire content, in each model, it is necessary to find an appropriate number of topics. By using topic numbers *k* ranging from 2 to 10, we initialized the LDA models and calculated the model coherence.

**Figure 7:**
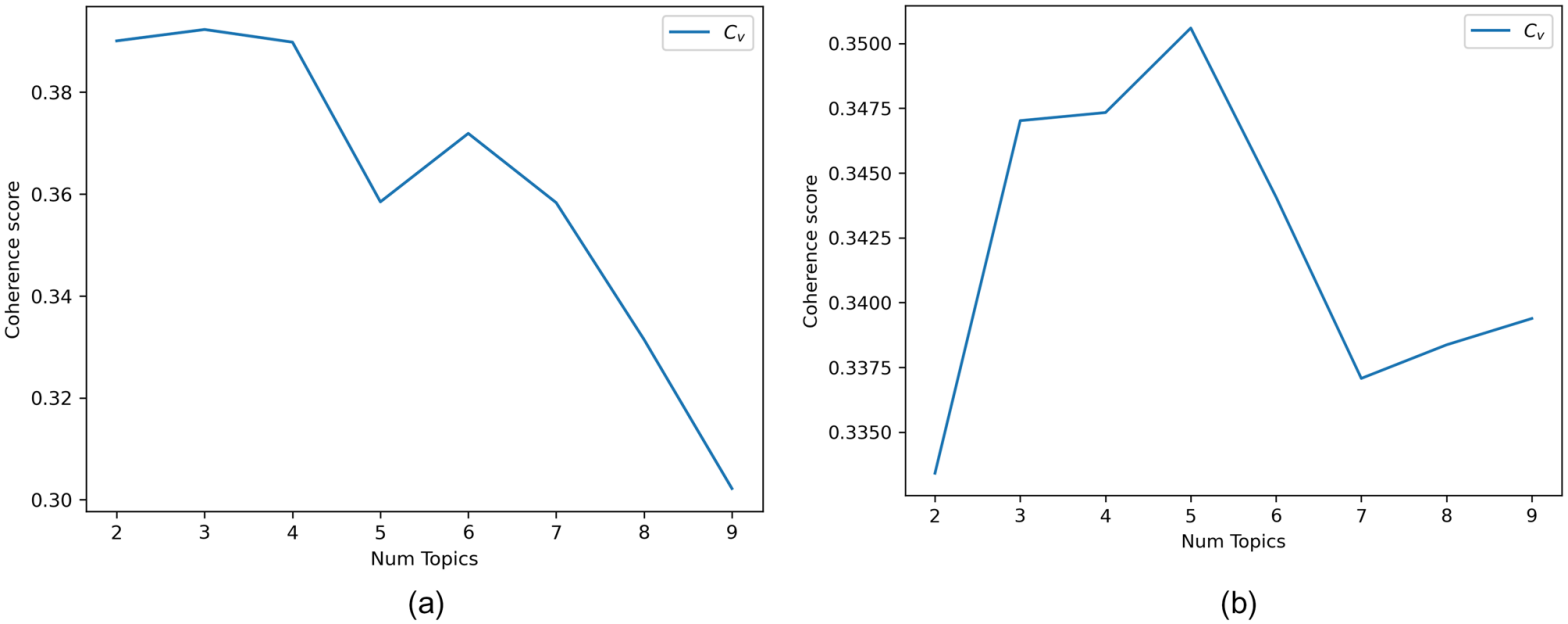
Coherence values of the LDA models.

We used $C_v$ coherence for both models as a first reference. This value indicates the degree of ‘sense’ and ‘cohesiveness’ of the main words within a topic.^63^

According to Fig. 7a, the coherence score peaked at 3 for the negative tweet model. In contrast, in the positive tweet model, the coherence score (Fig.7b) peaked at 3 and 5 topics. The choice of 5 topic numbers would lead to a nonuniform distribution on Principal Component (PC) axes displayed by pyLDAvis, which means that there is not a high degree of independence for each topic (see the LDAvis map in Supplementary Information ‘S2’ and ‘S3’). A good model is judeged by a higher coherence and an average distribution on the principal component analysis displayed by pyLDAvis.^52^ Therefore, we chose 3 as the topic number: the model has no intersections among topics, summarizes the whole word space well, and retains relatively independent topics.

The LDA analysis for negative and positive polarity tweets is shown in Table 4.

In the *negative* tweets, the first theme accounts for 37.5% of the total tokens and includes tokens such as ‘chatgpt’, ‘write’,‘art’, ‘need’, ‘thing’, ‘stop’ and ‘generate’. It is rather immediate to think that this negative opinion refers to tools such as ChatGPT and expresses concerns about its use in art and writing. People think that human creativity can generate art and literature rather than generative AI; for this reason, the use of generative tools in these contexts should be stopped. The second theme accounts for 33.3% of the total tokens and includes the words ‘bad’, ‘job’, ‘technology’, ‘scary’, ‘change’ and ‘learn’. We infer people’s fear of the changes that technology will bring in the world of job and learning. Several words, such as ‘kill’, ‘war’, ‘worry’ ,‘fear’, ‘fight’, ‘robot’ and ‘dangerous’, are mentioned in the third topic. This may indicate a sort of ‘apocalyptic fear’ that artificial intelligence could lead us to a new war and the end of humankind. For a map representation of the three topics, see the Supplementary Information ‘S1’.

In the *positive* tweets, the first theme accounts for 36.3% of the total tokens and includes tokens such as ‘learn’, ‘help’, ‘technology’, ‘student’ and ‘job’. Based on this, we inferred that people think that AI technologies have the potential to improve the job world and educational system. The second theme accounts for 34.7% of the total tokens, including the words ‘chatgpt’, ‘well’, ‘write’, ‘create’ and ‘ask’, showing people’s positive perception of AIs such as ChatGPT writing, asking questions and creating new solutions. After all, several words, such as ‘love’, ‘chatgpt’, ‘good’ ,‘answer’ and ‘believe’, are mentioned in the third topic. This indicates that people ‘believe in AI’ and trust tha AI, particularly ChatGPT provides good answers and solutions. For a map representation of the three topics, see the Supplementary Information ‘S2’.

**Table 4:** LDA results for sentiment polarity.

(a) Negative				
	Topic 1 (37.5%)	Topic 2 (33.3%)	Topic 3 (29.2%)	
1	chatgpt	time	want	
2	get	really	kill	
3	say	bad	war	
4	write	job	mean	
5	ask	world	worry	
6	make	technology	crazy	
7	use	bing	man	
8	think	new	end	
9	art	scary	fuck	
10	need	change	fear	
11	thing	much	fight	
12	people	understand	robot	
13	stop	model	dangerous	
14	answer	become	stupid	
15	generate	learn	play	

(b)Positive				
	Topic 1 (36.3%)	Topic 2 (34.7%)	Topic 3 (29.0%)	
1	learn	chatgpt	know	
2	great	get	love	
3	help	well	chatgpt	
4	technology	make	good	
5	thank	think	model	
6	human	new	check	
7	need	use	datum	
8	tool	write	answer	
9	student	create	power	
10	work	ask	sure	
11	today	time	already	
12	interesting	go	much	
13	way	say	application	
14	job	thing	web	
15	social	see	believe	

Percentage size and first 15 words of each topic.

Based on the LDA outputs, the following six topics were identified:

- For ‘negative polarity’ tweets:
  - Topic 1: Concerns about ChatGPT use in art and writing
  - Topic 2: Fear of changes in the world of job and learning
  - Topic 3: Apocalyptic-fear.

- For ‘positive polarity’ tweets:
  - Topic 1: AI technologies can improve job and learning
  - Topic 2: Useful ChatGPT features
  - Topic 3: Belief in the ability of ChatGPT.

## Limitations

This study has several limitations, which can be summarized as follows.

1. Limitations related to the use of keywords to extract tweets. Sometimes, keywords can be ambiguous, leading to noise-affected results. Due to the dynamic nature of social media, trends and topics change rapidly. Keywords might quickly lose relevance as new terms emerge.
2. Limitations related to *emotion analysis*. A first limitation is that the number of emotion categories was limited to 8;^51,66^ however, emotion is a broad concept and, according to Cowen and Keltner^67^, may involve up to 27 categories. A second limitation is that misspelled words could not be identified or analysed in the algorithm. Further limitations involve the dictionary of sentiments (“lexicon”) developed by Mohammad and Turney for emotion analysis.^51^ This dictionary maps a list of language features to emotion intensities, where:
  Only 5 individuals were recruited to annotate a term against each of the 8 primary emotions.
  The emotions associated with a term were annotated without considering the possible term context.
  Although the percentages of agreement were apparently high, interrater reliability statistics were not reported.
3. Limitations of topic analysis. Considering that LDA is an unsupervised learning technique, the main limitation is the degree of subjectivity in defining the topic created.^45^
4. Limitations of Twitter-based studies. Twitter data generally underestimate the opinions of people aged 50 and over, because approximately 83% of Twitter users worldwide are indeed under age 50.^49^ However, in the present study bias has an almost negligible impact: the future of AI-powered e-learning technologies indeed has a greater impact on younger people than on older people.

## Conclusions and future perspectives

With the aim of studying the opinions and emotions related to AI-powered e-learning technologies, we collected tweets on this issue and carried out a sentiment analysis using the VADER and *Syuzhet* packages in combination with a topic analysis.

There is no doubt that artificial intelligence has the potential to transform the whole education system. The results showed a predominance of positive attitudes: topics with a positive outlook indicate trust and hope in AI tools that can improve efficiency in jobs and the educational world. Indeed, among the eight emotions of the *Syuzhet* package, ‘trust’ and ’joy’ were the most positive emotions observed in the tweets, while ‘fear’ was the most common negative emotion. Based on the analysis, two particular aspects of fear were identified: an ‘apocalyptic fear’ that artificial intelligence could lead to the end of humankind and a fear of the ‘future of artistic and intellectual jobs’, as AI could not only destroy human art and creativity but also make individual contributions of students and researchers not assessable.

In our analysis, people with positive sentiments were directed towards ‘adaptive learning’, ‘social learning’ and ‘gamification’. Therefore, from a future perspective, we can expect an ever-increasing implementation of these aspects in e-learning. AI could help educators ‘adapt learning’ techniques to tailor them to the individual student, with his or her own interests, strengths and preferences.

By analysing data about interactions between students, AI can identify opportunities for collaboration between students and thus transform ‘social learning’ into ‘collaborative learning’. AI could help educators create more effective group work assignments, provide targeted support to struggling students, and promote positive social interactions among them.

In class, instead of administering boring tests, AI-powered ‘games and simulations’ could increasingly provide engaging and interactive learning experiences to help students develop skills and knowledge in a fun and engaging way. Moreover, gamification could be increasingly useful for providing immediate feedback and monitoring student progress over time.

Despite our analysis highlighting the great potential of and people’s expectations for AI-based technologies, there is an aspect that cannot be elucidated by examining tweets.

Algorithms such as ChatGPT disrupt traditional text-based assessments, as students can query the program to research a topic that results in documents authored by an algorithm and ready to submit as a graded assignment. Therefore, we need to reimagine student assessment in new ways. The current debate is whether educators should ban artificial intelligence platforms through school internet filters^38^ or embrace algorithms as teaching and research tools.^68^

In March, 2023, Italy was the first government to ban ChatGPT as a result of privacy concerns. The Italian data-protection authority said there were privacy concerns relating to the model and said it would investigate immediately. However, in late April, the ChatGPT chatbot was reactivated in Italy after its maker OpenAI addressed issues raised by Italy’s data protection authority.

Regardless of privacy concerns, possible data manipulations or the right answers to AI-based tools, we believe that the future cannot be stopped.

## Supplementary Information

Supplementary Figure S1.

Supplementary Figure S2.

Supplementary Figure S3.

Supplementary Table S1.

## Data Availability

All data generated or analyzed during this study are included in the Supplementary Information Files of this published article.
