# Supplementary material for: Twitter users perceptions of AI-based e-learning technologies
Source: Sci Rep. 2024 Mar 11;14:5927. doi: 10.1038/s41598-024-56284-y (PMC11639736; doi:10.1038/s41598-024-56284-y)
Supplement: Supplementary file 1 — Supplementary Figure S1. [file 41598_2024_56284_MOESM1_ESM.pdf]

Figure S1. LDAvis maps for the LDA topic model for 3 topic numbers. The model refers to tweets with *negative sentiment* and was obtained by selecting the threshold lambda=0.7. Each of the three images below depicts the first 30 words for the selected topic (circle displayed in red) in the image.

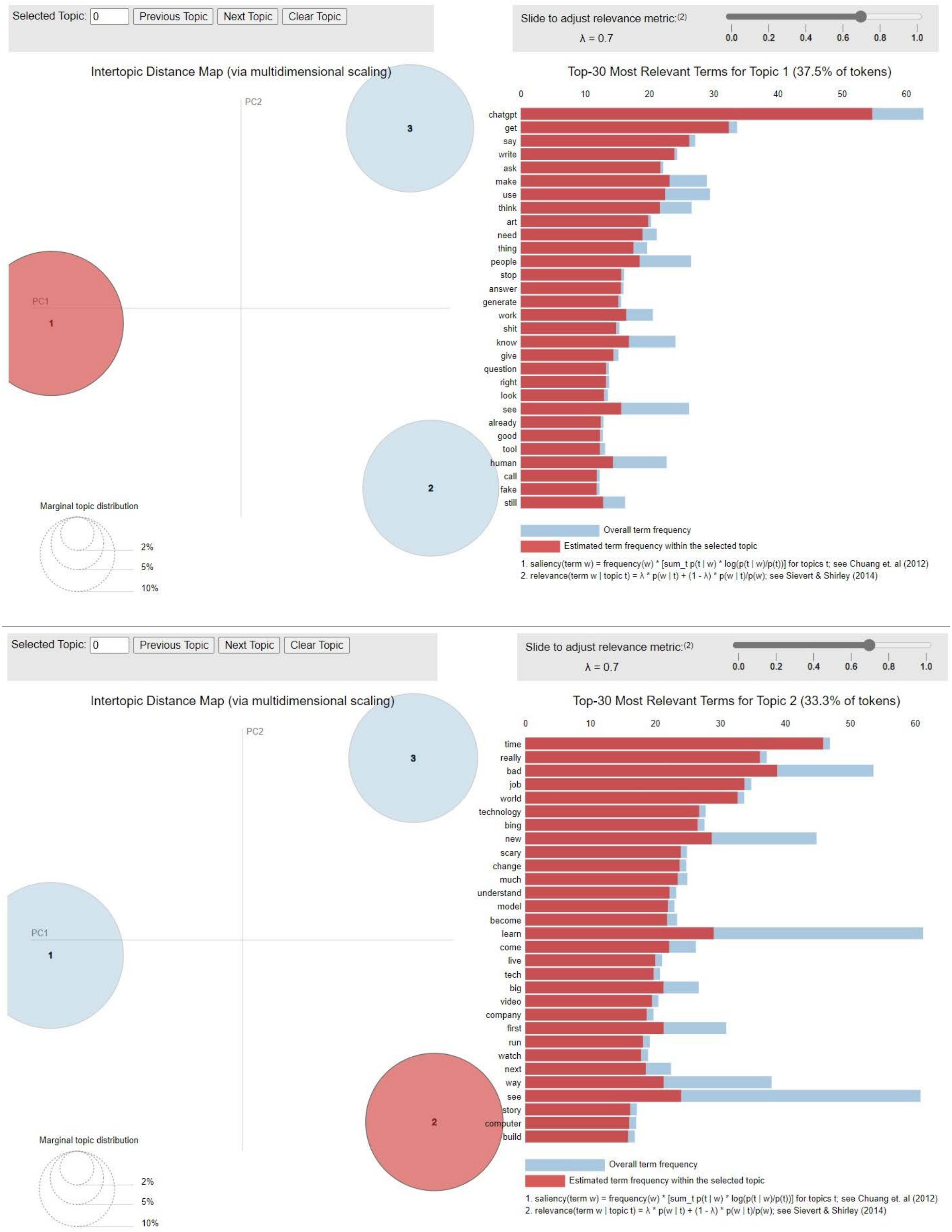

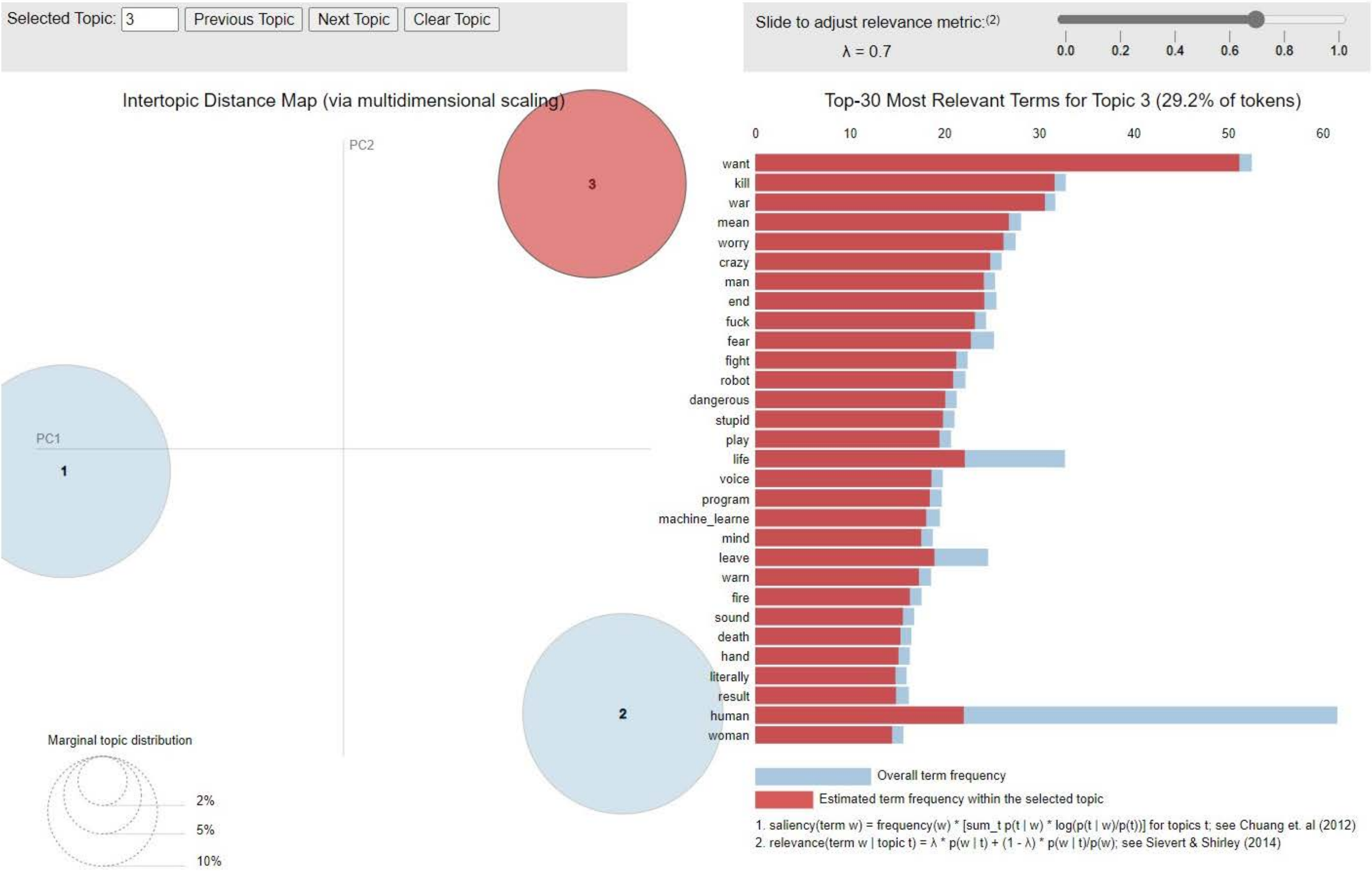
Selected Topic: 3
Previous Topic
Next Topic
Clear Topic
Slide to adjust relevance metric:(2)
λ = 0.7
0.0 0.2 0.4 0.6 0.8 1.0
Intertopic Distance Map (via multidimensional scaling)
PC2
PC1
1
2
3
Marginal topic distribution
2%
5%
10%
Top-30 Most Relevant Terms for Topic 3 (29.2% of tokens)
0 10 20 30 40 50 60
want
kill
war
mean
worry
crazy
man
end
fuck
fear
fight
robot
dangerous
stupid
play
life
voice
program
machine_learne
mind
leave
warn
fire
sound
death
hand
literally
result
human
woman
Overall term frequency
Estimated term frequency within the selected topic
1. saliency(term w) = frequency(w) * [sum_t p(t | w) * log(p(t | w)/p(t))] for topics t; see Chuang et. al (2012)
2. relevance(term w | topic t) = λ * p(w | t) + (1 - λ) * p(w | t)/p(w); see Sievert & Shirley (2014)
