# Supplementary material for: Twitter users perceptions of AI-based e-learning technologies
Source: Sci Rep. 2024 Mar 11;14:5927. doi: 10.1038/s41598-024-56284-y (PMC11639736; doi:10.1038/s41598-024-56284-y)
Supplement: Supplementary file 2 — Supplementary Figure S2. [file 41598_2024_56284_MOESM2_ESM.pdf]

Figure S2. LDAvis maps for the LDA topic model for 3 topic numbers. The model refers to tweets *with positive sentiment* and was obtained by selecting the threshold lambda=1. Each of the three images below depicts the first 30 words for the selected topic (circle displayed in red) in the image.

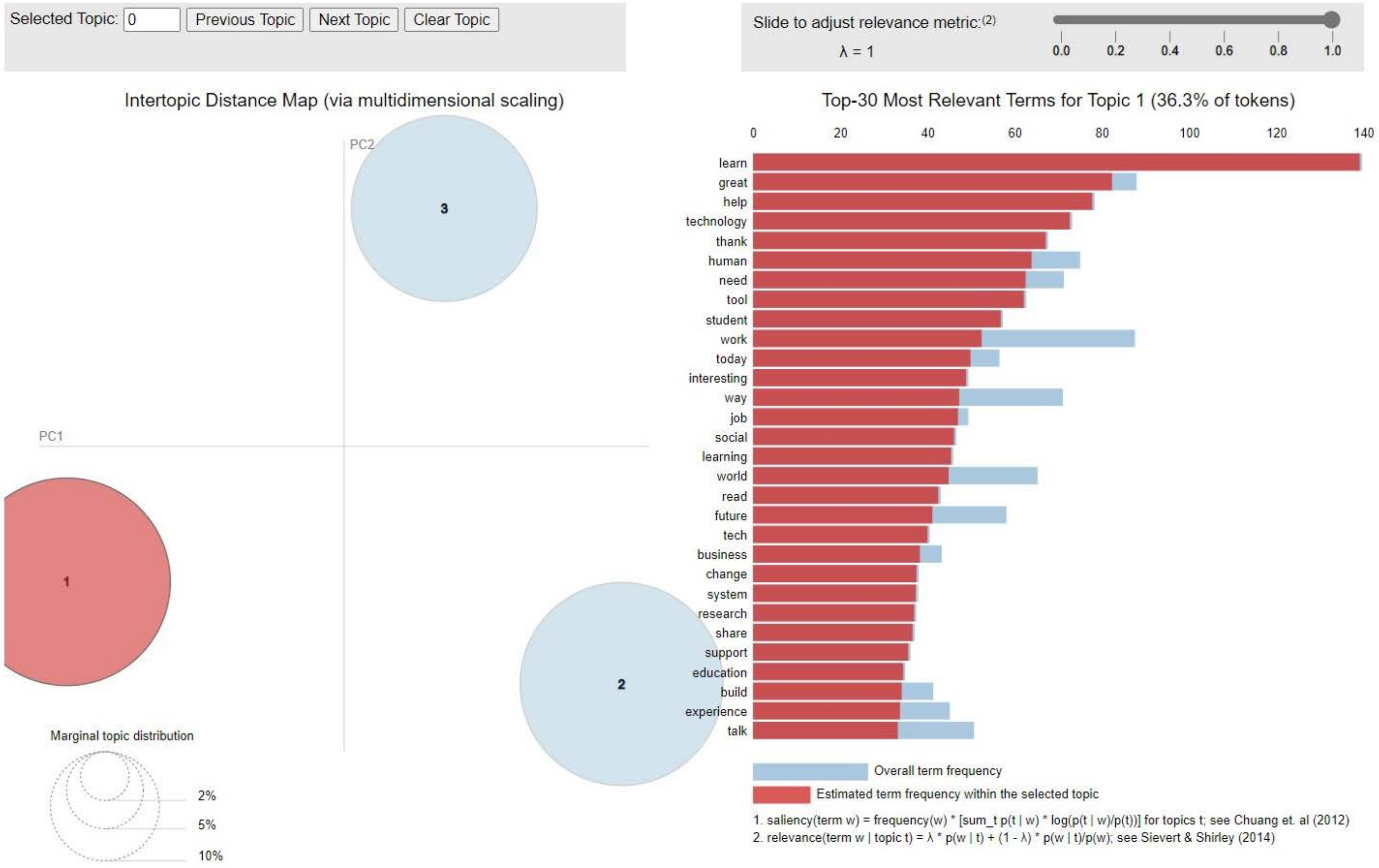

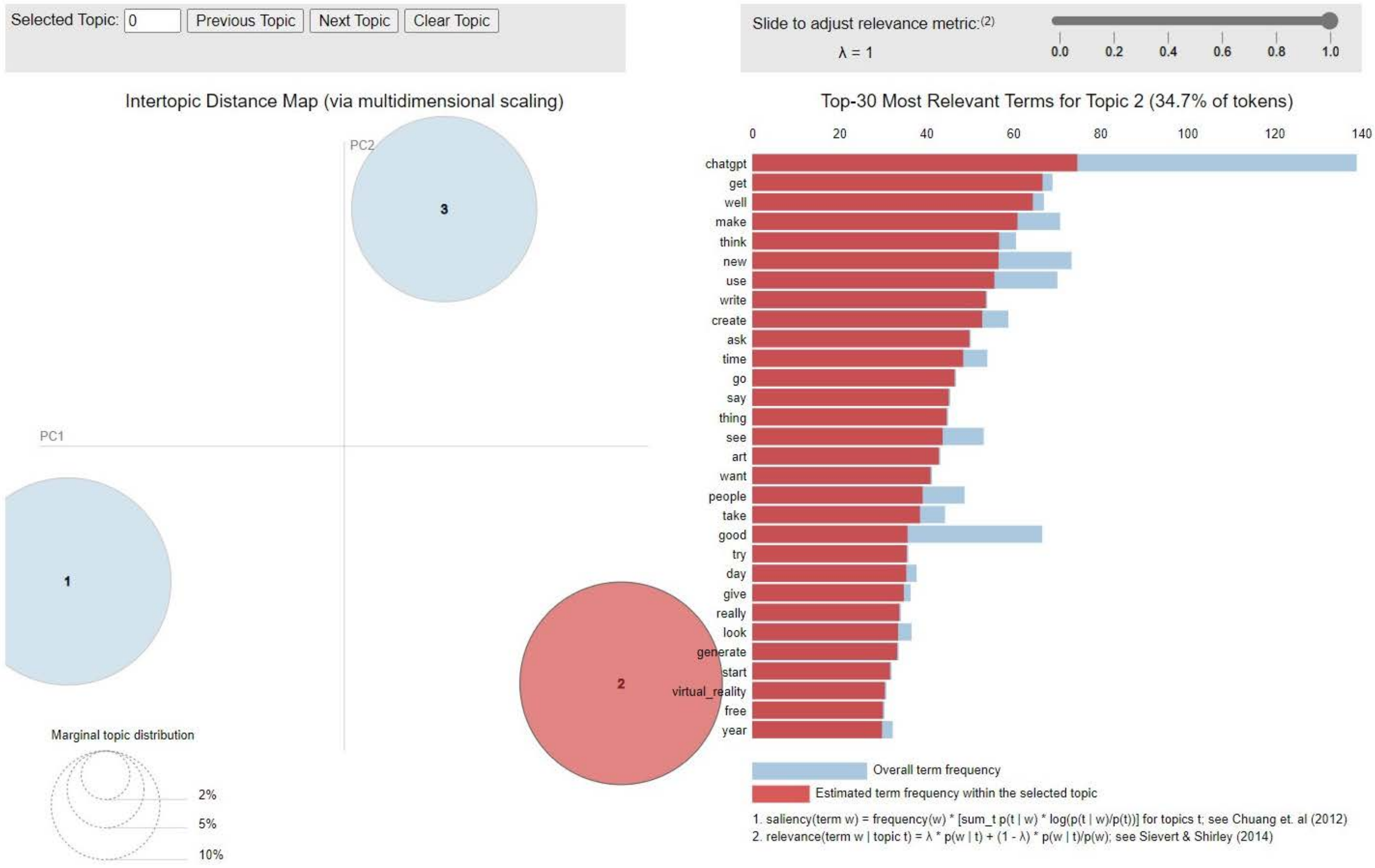

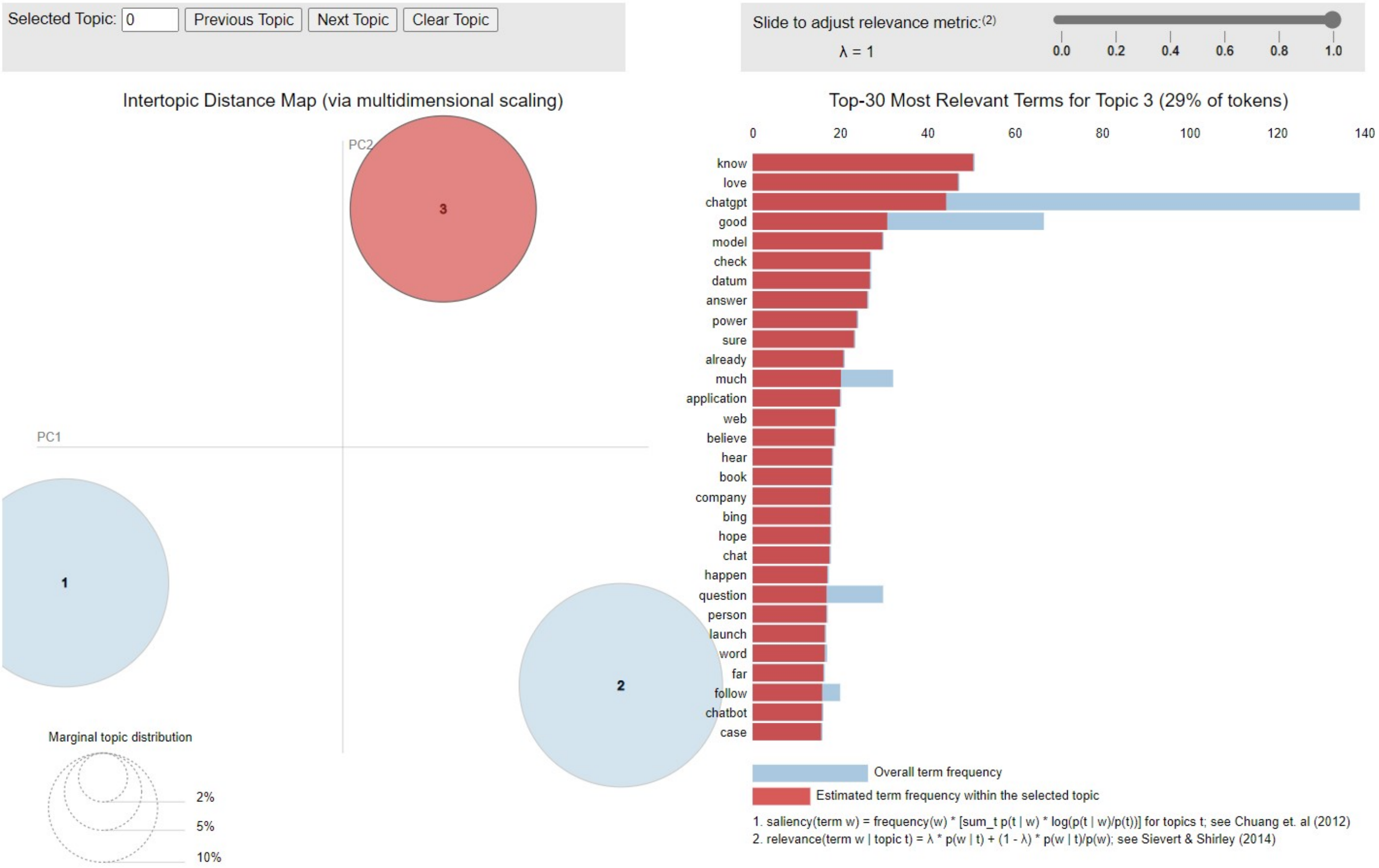

Selected Topic: 0
Previous Topic
Next Topic
Clear Topic
Slide to adjust relevance metric:(2)
λ = 1
0.0
0.2
0.4
0.6
0.8
1.0
Intertopic Distance Map (via multidimensional scaling)
PC2
PC1
3
1
2
Marginal topic distribution
2%
5%
10%
Top-30 Most Relevant Terms for Topic 3 (29% of tokens)
0
20
40
60
80
100
120
140
know
love
chatgpt
good
model
check
datum
answer
power
sure
already
much
application
web
believe
hear
book
company
bing
hope
chat
happen
question
person
launch
word
far
follow
chatbot
case
Overall term frequency
Estimated term frequency within the selected topic
1. saliency(term w) = frequency(w) * [sum_t p(t | w) * log(p(t | w)/p(t))] for topics t; see Chuang et. al (2012)
2. relevance(term w | topic t) = λ * p(w | t) + (1 - λ) * p(w | t)/p(w); see Sievert & Shirley (2014)
