# Supplementary material for: Twitter users perceptions of AI-based e-learning technologies
Source: Sci Rep. 2024 Mar 11;14:5927. doi: 10.1038/s41598-024-56284-y (PMC11639736; doi:10.1038/s41598-024-56284-y)

Figure S3. LDAvis map for LDA topic model for 5 topic numbers. The model refers to tweets with *positive sentiment*. As can be seen from the figure below, topics 2, 3, and 5 tend to overlap and cause some interpretation difficulties.

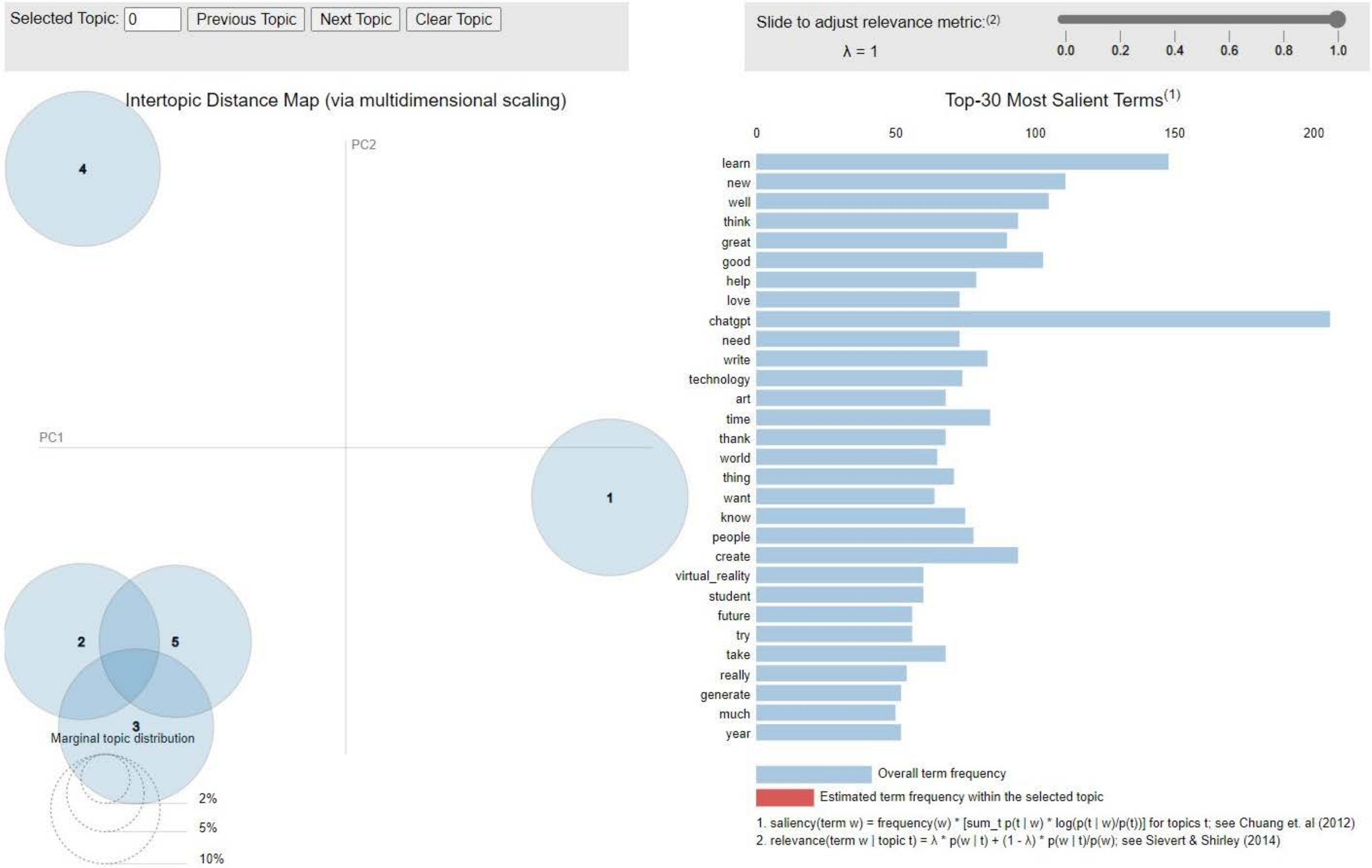

Supplement: Supplementary file 3 — Supplementary Figure S3. [file 41598_2024_56284_MOESM3_ESM.pdf]
